# A seven-gene prognostic model for platinum-treated ovarian carcinomas

**DOI:** 10.1038/bjc.2011.219

**Published:** 2011-06-07

**Authors:** R Sabatier, P Finetti, J Bonensea, J Jacquemier, J Adelaide, E Lambaudie, P Viens, D Birnbaum, F Bertucci

**Affiliations:** 1 Department of Molecular Oncology, Centre de Recherche en Cancérologie de Marseille (CRCM), UMR891 Inserm, Institut Paoli-Calmettes (IPC), 13009 Marseille, France; 2 Department of Medical Oncology, CRCM, UMR891 Inserm, Institut Paoli-Calmettes (IPC), 232 bd Sainte-Marguerite, 13009 Marseille, France; 3 Department of Biopathology, IPC, 13009 Marseille, France; 4 Department of Surgery, IPC, 13009 Marseille, France; 5 Université de la Méditerranée, 13284 Marseille Cedex 07, France

**Keywords:** ovarian cancer, gene expression profiling, prognosis

## Abstract

**Background:**

Prognosis of ovarian carcinoma is poor, heterogeneous, and not accurately predicted by histoclinical features. We analysed gene expression profiles of ovarian carcinomas to identify a multigene expression model associated with survival after platinum-based therapy.

**Methods:**

Data from 401 ovarian carcinoma samples were analysed. The learning set included 35 cases profiled using whole-genome DNA chips. The validation set included 366 cases from five independent public data sets.

**Results:**

Whole-genome unsupervised analysis could not distinguish poor from good prognosis samples. By supervised analysis, we built a seven-gene optimal prognostic model (OPM) out of 94 genes identified as associated with progression-free survival. Using the OPM, we could classify patients in two groups with different overall survival (OS) not only in the learning set, but also in the validation set. Five-year OS was 57 and 27% for the predicted ‘Favourable’ and ‘Unfavourable’ classes, respectively. In multivariate analysis, the OPM outperformed the individual current prognostic factors, both in the learning and the validation sets, and added independent prognostic information.

**Conclusion:**

We defined a seven-gene model associated with outcome in 401 ovarian carcinomas. Prospective studies are warranted to confirm its prognostic value, and explore its potential ability for better tailoring systemic therapies in advanced-stage tumours.

Ovarian carcinoma is the first death cause from gynaecological malignancy in western countries. Its poor prognosis is linked to late diagnosis, usually done at advanced stage, and to the development of chemoresistance. The classical therapeutic sequence combines maximal debulking surgery followed by adjuvant platinum and paclitaxel-based chemotherapy (Bristow *et al*, 2002; International Collaborative Ovarian Neoplasm Group, 2002; Ozols *et al*, 2003; Cannistra, 2004). Unfortunately, 20% of patients are refractory to chemotherapy, and >50% of those who achieve initial complete remission relapse and succumb from disease progression (McGuire *et al*, 1999). Overall survival (OS) is thus short (5-year OS: 30–40% for all stages) and has remained stable for two decades, notably in advanced stages (McGuire *et al*, 1999).

Ovarian carcinoma is clinically heterogeneous. Patients with morphologically similar, advanced-stage tumours display a broad range of clinical outcomes. Prognostic features, including patient's age, performance status, FIGO stage, histological tumour grade and subtype, and initial surgery results, are insufficient to capture the important individual variations in response to chemotherapy and survival. For example, it is impossible to predict which patients will benefit or not benefit from systemic first-line platinum/taxane-based chemotherapy. The consequence is that all women are given the same regimen although they will not display the same response and outcome.

This heterogeneous outcome suggests the existence of biologically different forms. Potential prognostic or predictive biomarkers, such as *TP53*, *MYC*, ABC transporters, *BCL2*, or *BRCA* genes (Williams *et al*, 2005; Kommoss *et al*, 2007; Walsh *et al*, 2008; Gadducci *et al*, 2009), have been identified. However, none has been validated for routine use. Large-scale RNA expression profiling has been used to find genes associated with response to chemotherapy (Spentzos *et al*, 2005; Bild *et al*, 2006; Lage and Denkert, 2007) or prognosis (Spentzos *et al*, 2004; Hartmann *et al*, 2005; Bonome *et al*, 2008) in ovarian carcinomas. However, these studies were relatively limited to small or median size populations, notably regarding the validation set.

Our objective was to identify, from primary ovarian cancer biopsies, a molecular predictor, associated with increased survival following platinum-based chemotherapy, and to validate its performances in a large panel of independent tumours.

## Materials and methods

### Samples selection

Pre-treatment ovarian cancer samples from 35 patients who underwent initial surgery followed by platinum-based chemotherapy were available for RNA profiling. They were collected at the Institut Paoli-Calmettes (IPC) between January 1994 and June 2007. Each patient gave written informed consent for molecular analysis. This study was approved by our institutional ethic committee. After removal, samples were macrodissected by pathologists and frozen within 30 min of removal. All profiled specimens were reviewed by a pathologist (JJ) before RNA extraction and contained >60% of tumour cells. After surgery, patients were treated using platinum-based chemotherapy according to standard guidelines.

### Gene expression profiling

Total RNA isolation was done with the All prep DNA/RNA kit (Qiagen, Valencia, CA, USA). RNA integrity was assessed by 2100 Agilent bioanalyser (Agilent, Palo Alto, CA, USA).

Gene expression analysis was done with Affymetrix Human Exon 1.0 ST arrays (Affymetrix, Santa Clara, CA, USA), as recommended by the manufacturer (http://www.Affymetrix.com).

We limited our expression analysis to gene level using only known and identified transcripts (Core library, Affymetrix). Analyses are described in Supplementary materials and methods available online. They include unsupervised and supervised approaches. Supervised analysis (see study flowchart, Supplementary Figure 1) aimed at identifying in the IPC set a multigene expression predictor for progression-free survival (PFS). First, Cox regression analysis identified genes whose expression (continuous variable) was associated with PFS (*P*⩽0.01, Wald's test). A median expression profile of progressive samples was computed from these differential genes. A correlation score (Pearson's coefficient) of each sample with this profile was computed and used to classify samples. Two groups of samples were thus defined: an ‘Unfavourable group’ defined by a positive score and a ‘Favourable group’ defined by a negative score. Second, we defined, from these differential genes, an optimal prognostic model (OPM). Recursive iterations were performed with a multivariate Cox model. Variables selection was done with an iterative method including two steps with leave-one-out cross-validation. The ‘Forward’ step identified the most significant variable to classify the tumours. If its significance rate was >1% and the resulting classification was better than the one from the previous model, the variable was kept. The ‘Backward’ step took out variables one after the other one from this new model in a reverse way and evaluated all possible combinations to choose the most valuable one. This step was repeated until the model could not be improved. Once the best model was defined (OPM), a prediction score, defined by a Cox resulting linear function, was then calculated for each sample, thus defining two classes: the ‘Unfavourable’ class with a positive score and the ‘Favourable’ class with a negative score.

To validate the predictive performances of the model in independent ovarian carcinoma samples, we analysed five publicly available data sets (Berchuck *et al*, 2005, 2009; Partheen *et al*, 2006; Tothill *et al*, 2008; Denkert *et al*, 2009). We first identified the common genes. Then, we median centred the corresponding gene expression values within each data set (Berchuck's sets were pooled and doubloons were excluded). The prediction score (OPM) defined two classes: ‘Unfavourable’ (positive score) and ‘Favourable’ (negative score). Regarding the prognostic analysis, the clinical outcome available in these studies was OS. The value of time to death was available in three studies, but not in the two other studies where information was ‘Long survivors’ if OS was superior to 7 years and ‘Short survivors’ if inferior to 3 years in one study (Berchuck *et al*, 2005), and OS lower or higher than 5 years in the other one (Partheen *et al*, 2006).

### Statistical analysis

All statistical analyses were done in R version 2.6.1 (http://cran.r-project.org) and its associated packages. Details about clinical definitions and statistical analyses are given in Supplementary materials and methods.

## Results

### Gene expression profiling of ovarian carcinoma

We profiled 35 ovarian cancer samples from patients who underwent oophorectomy at the IPC. All cases were adenocarcinomas treated with platinum-based chemotherapy after surgery. Their characteristics are summarised in Table 1, and detailed in Supplementary Table 1. Most cases were serous, high-grade tumours, and advanced stages. Surgery was optimal for 46% of cases. After chemotherapy, the rate of clinical response was 68% (complete 57% and partial 11%). The rate of pathological complete response (pCR) was 57%. With a median follow-up of 55 months after diagnosis, 24 patients experienced disease progression (median PFS: 13 months and 5-year PFS: 20%) and 19 patients died (median OS: 37.5 months and 5-year OS: 37%).

**Figure 1:**
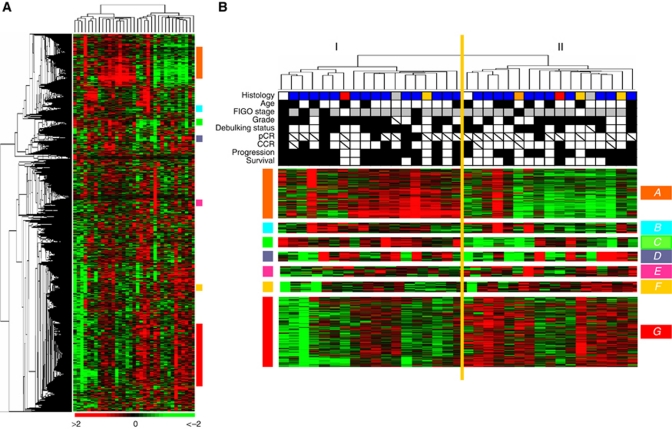
Gene expression unsupervised analysis of the 35 platinum-treated primitive ovarian carcinomas. (**A**) Thumbnail of the hierarchical clustering of the 35 platinum-treated ovarian carcinomas (columns) and the 4824 most variable genes (rows). According to a log_2_ pseudocolour scale, red indicates a high level of mRNA expression compared with the median value of a given gene, whereas green indicates a low level of expression. The magnitude of deviation from the median is represented by the colour saturation. The dendrogram of samples (above matrixes) represents overall similarities in gene expression profiles and is zoomed in (**B**). Coloured bars to the right indicate the locations of seven gene clusters of interest that are zoomed in (**B**). (**B**) Sample dendrogram with main gene clusters. Two large groups of samples (designated I and II) are evidenced by clustering and delimited by the orange vertical line. Under the dendrogram are noted the main histoclinical features coloured as below: *Histology*: blue, serous ADK; orange, mucinous; red, clear cells; yellow, endometrioid; grey, undifferentiated; white, mixed. *Age*: white <60 years; black >60 years. FIGO stage: white, I; grey, III; black, IV. *Grade*: white, 1–2; black, 3. *Debulking status*: white, optimal; black, suboptimal. *pCR and CCR*: white, yes; black, no. *Progression*: white, no; black, yes. *Survival*: white, alive; black, dead. Hatched squares, data not available. The colour reproduction of this figure is available at the *British Journal of Cancer* journal online.

Unsupervised analysis based on hierarchical clustering distinguished two groups of samples without any significant correlation with histoclinical data (Figure 1), and specifically clinical outcome. Kaplan–Meier analyses showed *P*-values of 0.88 and 0.09 for PFS and OS, respectively. Consistent with previous studies (Schaner *et al*, 2003), we identified coherent gene clusters involving in a specific biological function or chromosomal location. Seven of them are shown in Figure 1. Two of them included genes involved in stromal environment and cellular movement. The first one (cluster A) contained genes coding for proteins of the extracellular matrix (*COL1A1*, *COL1A2*, *FN1*, *VIM*, and *MMP2*) or involved in cellular mobility (*MYH11*, *MYL9*, and *MYLK*). The second one (cluster F) included genes coding for proteins involved in cellular adhesion, such as the claudins (*CLDN3*, *CLDN4*, and *CLDN7*) or *CDH1*. It is of note that expressions of these clusters were anti-correlated, suggesting that they may represent the two opposite sides of a same mechanism. Two other clusters were associated with immune response: cluster C was associated with the complement pathway (*C1QA*, *C1QB*, *C2*, *C3*, and *CFB*), the class II major histocompatibility complex (*DQA2*, *DRA*, and *DMA*) and the Natural Killer lymphocytes pathway (*FCER1G*, *LAIR1*, and *TYROBP*); cluster D contained genes linked to the Interferon pathway (*IFI6*, *IFI27*, and *IRF9*). We observed also a cluster linked to the 17q12 chromosomal region (cluster E), including *ERBB2* and neighbour genes (*TMEM99*, *PERLD1*, *C17orf157*). Cluster B contained genes involved in early cell response (*EGR*, *JUNB*, and *FOS*). Finally, several genes involved in cell-cycle control pathways such as DNA damage repair (*BARD1*, *FANCF*, *E2F3*, and *E2F5*), checkpoint control (*CHEK1*, *CCNB1*, *CCNB2*, *TOP2A*, and *TOP2B*), and apoptosis (*BAK1*, *CASP2*, *CASP8*, *CDC2*, and *MAPK8*) were in cluster G.

### Identification of a multigene predictor of survival

**Figure 2:**
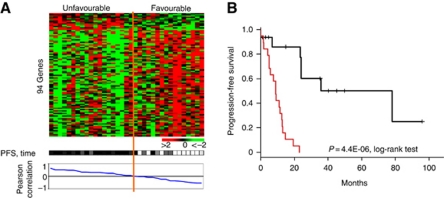
Supervised classification based on progression-free survival (Cox univariate analysis). (**A**) Expression plot of the 94 genes correlated with PFS. *Top panel*: *Rows*: normalised gene expression levels (legend similar to Figure 1A). Genes are classified from top to bottom according to their correlation with progression. *Columns*: samples are classified from left to right according to the correlation between their gene expression profile and the median expression profile of progressive samples, thus defining two groups ‘Unfavourable’ and ‘Favourable’. *Bottom panel*: progression status: white, no progression; for patients with disease progression, colour gradient according to PFS: dark black when early progression to clear grey when very late progression (here 80 months). (**B**) Association between the two gene expression groups identified by Pearson's correlation and PFS.

We then searched for a multigene expression predictor for PFS. Progression-free survival was correlated with mRNA expression of 94 genes identified by a Cox model (*P*⩽0.01), including 13 genes overexpressed and 81 genes underexpressed in early progressive samples (Supplementary Table 2). Figure 2 shows the classification of the 35 cases according to this gene expression signature. The two groups identified showed PFS difference (*P*=4.44E-06, log-rank test).

**Figure 3:**
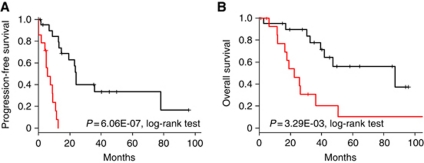
Survival of the two classes defined by our seven-gene OPM in the IPC learning set. (**A**) Progression-free survival. (**B**) Overall survival.

We then sought to define, among those 94 genes, an OPM with fewer genes potentially more easily applicable in clinical practice. Multivariate Cox analysis retained a seven-gene model (Supplementary Table 3), including two genes (*A1BG* and *PAH*) associated with unfavourable outcome and five genes (*SLC7A2*, *ALCAM*, *TMPRSS3*, *TSPAN6*, and *C14orf101*) associated with favourable outcome. Using a linear predictor, we defined two classes of tumours as ‘Favourable’ (*n*=21; 60%) and ‘Unfavourable’ (*n*=14; 40%). As expected, they displayed different clinical outcomes (Figure 3) with respective 2-year PFS equal to 46 and 0% (*P*=6.06E-07, log-rank test) and respective 2-year OS equal to 90 and 46% (*P*=3.29E-03, log-rank test).

Next, we analysed correlations between these two classes identified by our OPM and histoclinical features of samples: age, histological subtype, grade, stage, taxane use, surgical status, and pathological and clinical responses. As shown in Table 2A, we found no correlation except with survival. However, the rate of clinical complete response (CCR) was higher in the ‘Favourable’ class than in the ‘Unfavourable’ class (82 *vs* 46%), suggesting that the prognostic value of the OPM might be partly related to some predictive value for response to chemotherapy. Nevertheless, the OPM seemed to have a prognostic value within the subset of patients with CCR. In this subset, ‘Favourable’ cases had a 2-year and a 5-year PFS of 55% and 38%, respectively, *vs* 0% in the ‘Unfavourable’ group (*P*=2.56E-05, log-rank test). Thus, our model was able to identify poor-prognosis cases among those presenting the same response after treatment, suggesting a prognostic value linked to disease natural evolution, independent from the response to chemotherapy.

Then, we confronted our OPM to classical prognostic factors regarding the association with PFS: age, grade, stage, taxane use, and surgical status (Table 3A). Univariate analysis showed that FIGO stage, surgical status, and the OPM were correlated with PFS. In multivariate analysis, the OPM remained significant (*P*=2.2E-03, Wald's test), together with the FIGO stage, while the surgical status lost its prognostic value.

### External validation of the OPM

We sought to demonstrate the robustness and prognostic independence of our OPM in an independent validation set. We collected and pooled data from five recent prognostic studies of ovarian carcinomas, including 366 advanced stage tumours (FIGO stages III and IV). Their characteristics are resumed in Table 4. The clinical endpoint available through the five series was OS: the information (death or alive) was available for all 366 patients, with survival times mentioned for 262 patients. A total of 172 out of 366 women died from disease. For the 262 cases with reported survival times, 112 died and 150 remained alive with a median follow-up of 32 months (range 1–166) and a 5-year OS equal to 39% (31–49%).

**Figure 4:**
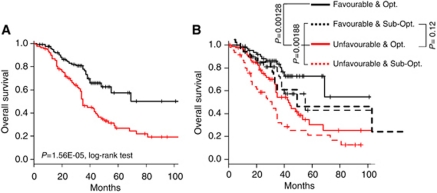
Survival of the two classes defined by our seven-gene OPM in the public validation set. (**A**) OS for the 262 patients with survival time available. (**B**) Similar to (**A**) with stratification based on the residual disease after surgery (Opt., optimal surgery; Sub-Opt., suboptimal surgery). *P*-values were assessed with the log-rank test.

Applied to these samples, the seven-gene OPM defined a ‘Favourable’ class (*n*=187; 51%) and an ‘Unfavourable’ class (*n*=179; 49%) that strongly correlated with survival. The ‘Favourable’ class contained 121 of the 194 alive patients and the ‘Unfavourable’ class contained 106 of the 172 deceased patients (Se=61.6%, Sp=62.4%, odd ratio=2.7, 95% CI=1.7–4.2; *P*=6.1E-06, Fisher's exact test), thus confirming the prognostic value of our OPM in a large and independent data set. In this validation set (Table 2B), patients classified as ‘Favourable’ were slightly younger (*P*=0.02) and had a better OS than ‘Unfavourable’ cases with 5-year OS of 57 *vs* 27%, respectively (*P*=1.56E-05; Figure 4A).

In this data set, univariate analysis for OS retained as significant the same features as in the learning set, that is, the OPM-based classification, as well as the classical FIGO stage and the amount of residual disease after surgery (Table 3B). In multivariate analysis, our seven-gene model and the FIGO stage remained significant, further underlining the robustness of the model and its capacity to predict clinical outcome independently of other clinical features and better than the residual disease after surgery. Indeed, OS was higher in the ‘Favourable’ cases than in the ‘Unfavourable’ cases, both in tumours without residual disease (5-year OS: 73 *vs* 30% *P*=1.3E-03) and in tumours with residual disease after surgery (5-year OS: 43 *vs* 21%, *P*=1.9E-03; Figure 4B). Moreover ‘Favourable’ cases with residual disease after surgery displayed longer survival than tumours without residual disease but with an ‘Unfavourable’ profile (*P*=0.12), even if the difference was not significant.

## Discussion

Despite frequent initial chemosensitivity, the prognosis of advance-stage ovarian cancer is poor with a long-term OS of 25%. Classical prognostic criteria are insufficient to accurately predict the survival of an individual patient, and need to be improved. Response to chemotherapy is an imperfect prognostic factor as it correlates more with immediate clinical outcome than with long-term PFS and OS, which depend on additional factors such as the invasive potential and growth of the tumour. Early identification of the ∼80% of patients who will die from disease progression despite the initial response to standard treatment is crucial. It should help guide initial therapy by using experimental approaches such as novel first-line drugs, novel strategies such as intra-peritoneal chemotherapy or maintenance chemotherapy, or by using existing alternative chemotherapy regimens instead of the standard regimen. Some gene profiling studies have addressed the issue of survival prediction in ovarian cancer (for review, see Sabatier *et al* (2009)). In most of them, however, the validation of the multigene predictor was either absent or done on a relatively small validation set, inferior to 118 samples for the largest (Denkert *et al*, 2009).

Using whole-genome DNA microarrays, we profiled a unicentric series of 35 pre-treatment platinum-treated ovarian carcinomas, including a majority of advanced stages. Supervised analysis of gene expression data identified 94 genes whose expression was correlated with PFS, including genes involved in DNA repair (*APEX1*, *WDR6*, and *PARP2*) and apoptosis (*CCAR1*, *CASP2*, *IKBKB*, and *PDCD6IP*), or known to be associated with malignancy (*S100A8*, *FNTA*, and *CLUAP1*). It is of note that most of these 94 genes were not present in previously published signatures. This discrepancy between prognostic gene signatures identified using high-throughput technologies has already been reported in several cancers, notably breast (Bertucci *et al*, 2006) and ovarian (Sabatier *et al*, 2009) cancers. It can be explained not only by the technological and methodological differences between these studies, but also by the relatively small size of populations analysed and the patients’ heterogeneity, both in term of clinical and pathological features definitions. In this context, the validation of a signature in large and independent series is crucial to confirm its robustness.

From this 94-gene list, we established a seven-gene OPM able to classify, independently from classical prognostic features, our samples in two classes with different clinical outcome: a ‘Favourable’ with 5-year OS of 56%, and an ‘Unfavourable’ class with 5-year OS of 10%. Importantly, when applied to a large independent validation set (*N*=366), which represents so far the largest one reported in the literature, our model maintained its strong and independent prognostic value in multivariate analysis with 5-year OS of 57% in the ‘Favourable’ class and 27% in the ‘Unfavourable’ class.

Whether our model reflects the chemosensitivity of the tumour and/or its metastatic and proliferative potential cannot be determined, but interestingly, it remained predictive of survival when applied to the homogeneous respective groups of patients with CCR to chemotherapy, suggesting it is partly independent of chemosensitivity. The two genes of the model associated with poor prognosis; *A1BG* and *PAH,* are known to be involved in cancer and particularly in ovarian neoplasm development. Phenylalanine hydroxylase concentrations were higher in patients with advanced-stage disease (Neurauter *et al*, 2008) and correlated with concentrations of immune markers (tumour necrosis factor-*α* receptor and neopterin). Alpha-1 *β* glycoprotein (A1BG), a secreted protein of unknown function, was underexpressed in urines of bladder cancer patients (Kreunin *et al*, 2007). It presents several similarities with its opossum homologue, Oprin (Catanese and Kress, 1992). Oprin has a metalloproteinase inhibitor function and is similar to TIMP (tissue inhibitor of metalloproteinase), which can have a role in angiogenesis, cellular proliferation, and tumour progression (Chirco *et al*, 2006). Expression of A1BG is also stronger in pancreatic cancer than in normal pancreas (Tian *et al*, 2008). Three of the five genes of our model correlated with good prognosis are implicated in oncogenesis. *TMPRSS3* is overexpressed in pancreatic cancers when compared with normal pancreatic and pancreatitis tissues (Wallrapp *et al*, 2000). It is overexpressed in ovarian carcinomas as compared with non-malignant tissues (Sawasaki *et al*, 2004), but its prognostic value has never been evaluated. ALCAM protein has been associated with prognosis in melanoma (van Kilsdonk *et al*, 2008), ovarian (Mezzanzanica *et al*, 2008), breast (Ihnen *et al*, 2010), prostate (Kristiansen *et al*, 2005), colorectal (Weichert *et al*, 2004), and pancreatic (Kahlert *et al*, 2009) cancers. *SLC7A2* expression is higher in oestrogen receptor (ER)-positive breast tumours than in ER-negative ones (Tozlu *et al*, 2006).

In *conclusion*, we have developed, and validated in a large series of samples, a seven-gene model associated with survival of platinum-treated ovarian carcinoma patients. If further retrospective and prospective validation studies confirm its relevance, our model could help tailor the systemic treatment of advanced-stage ovarian cancer. Based on their low likelihood of achieving prolonged survival with standard first-line platinum-based therapy, the ‘Unfavourable’ patients might be guided, at the time of diagnosis, towards investigational treatment approaches to be defined. Furthermore, a better understanding of the implication in ovarian oncogenesis of the genes present in our model might help develop alternative therapies.

## Figures and Tables

**Table 1:** Histoclinical features of the IPC learning set (*N*=35)

*Age (years)*	
Median	57
Range	26–83
	
*Histological subtype*	
Serous	25 (71%)
Endometrioid	3 (9%)
Clear cells	2 (6%)
Mucinous	1 (3%)
Mixed	2 (6%)
Undifferentiated	2 (6%)
	
*FIGO stage*	
I	2 (6%)^a^
III	27 (77%)
IV	6 (17%)
	
*Histological grade*	
1	1 (3%)
2	9 (26%)
3	24 (69%)
NI	1 (3%)
	
*Surgical status*	
Optimal surgery	16 (46%)
Nor performed or suboptimal	18 (51%)
NA	1 (3%)
	
*Clinical response*	
Complete response	20 (57%)
Partial response	4 (11%)
Stable disease	4 (11%)
Progression	2 (6%)
NA	5 (14%)
	
*Pathological response*	
Complete	8 (57%)^b^
Non complete	6 (43%)^c^
	
Median follow-up, months (95% CI)	55 (34–76)
Median PFS, months (95% CI)	13 (10.2–15.7)
Five-year PFS (95% CI)	20% (10–41)
Median OS, months (95% CI)	38 (25–50)
Five-year OS (95% CI)	37% (22–61)

Abbreviations: CI=confidence interval; FIGO=Fédération Internationale de Gynécologie et Obstétrique; IPC=Institut Paoli-Calmettes; NA=not assessable; NI=not indicated; OS=overall survival; PFS=progression-free survival.

a One clear cell carcinoma+one grade 3 endometrioid carcinoma.

b All with complete clinical response after chemotherapy.

c Three patients with complete clinical response, one with partial clinical response, and two with stable disease.

**Table 2:** Comparison of the two classes defined by the OPM

**A: IPC learning set (*N*=35)**				
	***N***	**Favourable (*N*=21)**	**Unfavourable (*N*=14)**	***P*-value**
Age (years)	35	54 (26–74)	62.5 (41–83)	0.08^a^
				
*Histology*				0.70^b^
Others	10	7 (33%)	3 (21%)	
Serous	25	14 (67%)	11 (79%)	
				
*Grade*				0.14^b^
1+2	10	8 (40%)	2 (14%)	
3	24	12 (60%)	12 (86%)	
				
*FIGO stage*				0.66^b^
⩽III	29	18 (86%)	11 (79%)	
IV	6	3 (14%)	3 (21%)	
				
*Taxane*				0.28^b^
Yes	24	16 (76%)	8 (57%)	
No	11	5 (24%)	6 (43%)	
				
*Optimal debulking*				1^b^
Yes	16	10 (48%)	6 (46%)	
No	18	11 (52%)	7 (54%)	
				
*Pathological response*				0.24^b^
Complete	8	7 (70%)	1 (25%)	
No complete	6	3 (30%)	3 (75%)	
				
*Clinical response*				0.06^b^
Complete	20	14 (82%)	6 (46%)	
No complete	10	3 (18%)	7 (54%)	
Two-year OS	35	90%	46%	**3.29E-03** c
Two-year PFS	35	46%	0%	**6.06E-07** c
				
**B: public validation set (*N*=366)**				
	***N***	**Favourable (*N*=187)**	**Unfavourable (*N*=179)**	***P*-value**
Age, median (years)	295	58.5 (33–79)	62 (33–84)	**0.02** a
				
*Grade*				0.46^b^
1+2	148	79 (43%)	69 (39%)	
3	215	106 (57%)	109 (61%)	
				
*FIGO stage*				0.08^b^
III	337	177	160	
IV	29	10	19	
				
*Residual disease*				0.59^b^
Yes	153	81 (47%)	72 (43%)	
No	187	93 (53%)	94 (57%)	
				
Five-year OS^d^	262	57%	27%	**1.56E-05** c

Abbreviations: CCR=clinical complete response; FIGO=Fédération Internationale de Gynécologie et Obstétrique; IPC= Institut Paoli-Calmettes; *N*=number of samples with data available; pCR=pathological complete response; PFS=progression-free survival; OPM=optimal prognostic model; OS=overall survival.

a Mann–Whitney test.

b Fisher's exact test.

c Log-rank test.

d Survival time was available for 120 patients in the ‘Favourable’ group and 142 patients in the ‘unfavourable’ group. Signficant *P*-values were highlighted with bold characters.

**Table 3:** Univariate and multivariate analyses for survival

	**Cox univariate**				**Cox multivariate**			
	***N*** a	**Hazard ratio**	**95% CI**	***P*-value**	***N*** a	**Hazard ratio**	**95% CI**	***P*-value**
*A: IPC set, PFS analysis*								
Age (years)	35	1.02	0.98–1.05	0.36				
Grade 3 (*vs* 1+2)	34	1.42	0.59–3.43	0.43				
FIGO stage (IV *vs* others)	35	22.9	5.16–101	**3.80E-05**	34	8.34	1.93–36.1	**4.60E-03**
Taxane without (*vs* with)	35	1.83	0.82–4.07	0.14				
Non-optimal surgery (*vs* optimal)	34	3.35	1.34–8.38	**9.80E-03**	34	2.36	0.89–6.26	0.09
OPM Unfavourable (*vs* Favourable)	35	14.3	3.87–53.1	**6.80E-05**	34	8.59	2.17–34.0	**2.20E-03**
								
*B: Validation set, OS analysis*								
Age (years)	191	1.01	0.99–1.04	0.23				
Grade 3 (*vs* 1+2)	260	1.01	0.69–1.50	9.50E-01				
FIGO stage (IV *vs* III)	262	2.87	1.73–4.77	**4.71E-05**	236	2.60	1.48–4.60	**9.60E-04**
Residual disease (present *vs* absent)	235	1.59	1.07–2.35	**2.2E-02**	236	1.47	0.97–2.22	0.07
OPM Unfavourable (*vs* Favourable)	262	2.42	1.6–3.66	**2.72E-05**	236	2.56	1.62–4.03	**5.40E-05**

Abbreviations: 95% CI=95% confidence interval; FIGO=Fédération Internationale de Gynécologie et Obstétrique; OPM=optimal prognostic model; OS=overall survival; PFS=progression-free survival.

a Number of patients with data available. Signficant *P*-values were highlighted with bold characters.

**Table 4:** Histoclinical features of the validation set (*n*=366)

*Age (years)* a	
Median	60
Range	33–84
	
*Histological subtype*	
Serous	351 (96%)
Endometrioid	11 (3%)
Others	4 (1%)
	
*FIGO stage*	
III	337 (92%)
IV	29 (8%)
	
*Histological grade*	
1	16 (4%)
2	132 (36%)
3	215 (59%)
NI	3 (1%)
	
*Residual disease after surgery*	
Yes	187 (51%)
No	153 (42%)
NI	26 (7%)
	
Median follow-up (months)^b^	32
Number of deaths	172 (112 with time of death available)
Five-year OS (95% CI)^b^	39% (31–49)

Abbreviations: CI=confidence interval; FIGO=Fédération Internationale de Gynécologie et Obstétrique; NI=not indicated; OS=overall survival.

a 295 samples with data available.

b For the 262 cases with follow-up time available.
