# Supplementary material for: A seven-gene prognostic model for platinum-treated ovarian carcinomas
Source: Br J Cancer. 2011 Jun 7;105(2):304–11. doi: 10.1038/bjc.2011.219 (PMC3142802; doi:10.1038/bjc.2011.219)
Supplement: Supplementary Figure 1 [file bjc2011219x1.ppt]

## Slide 1
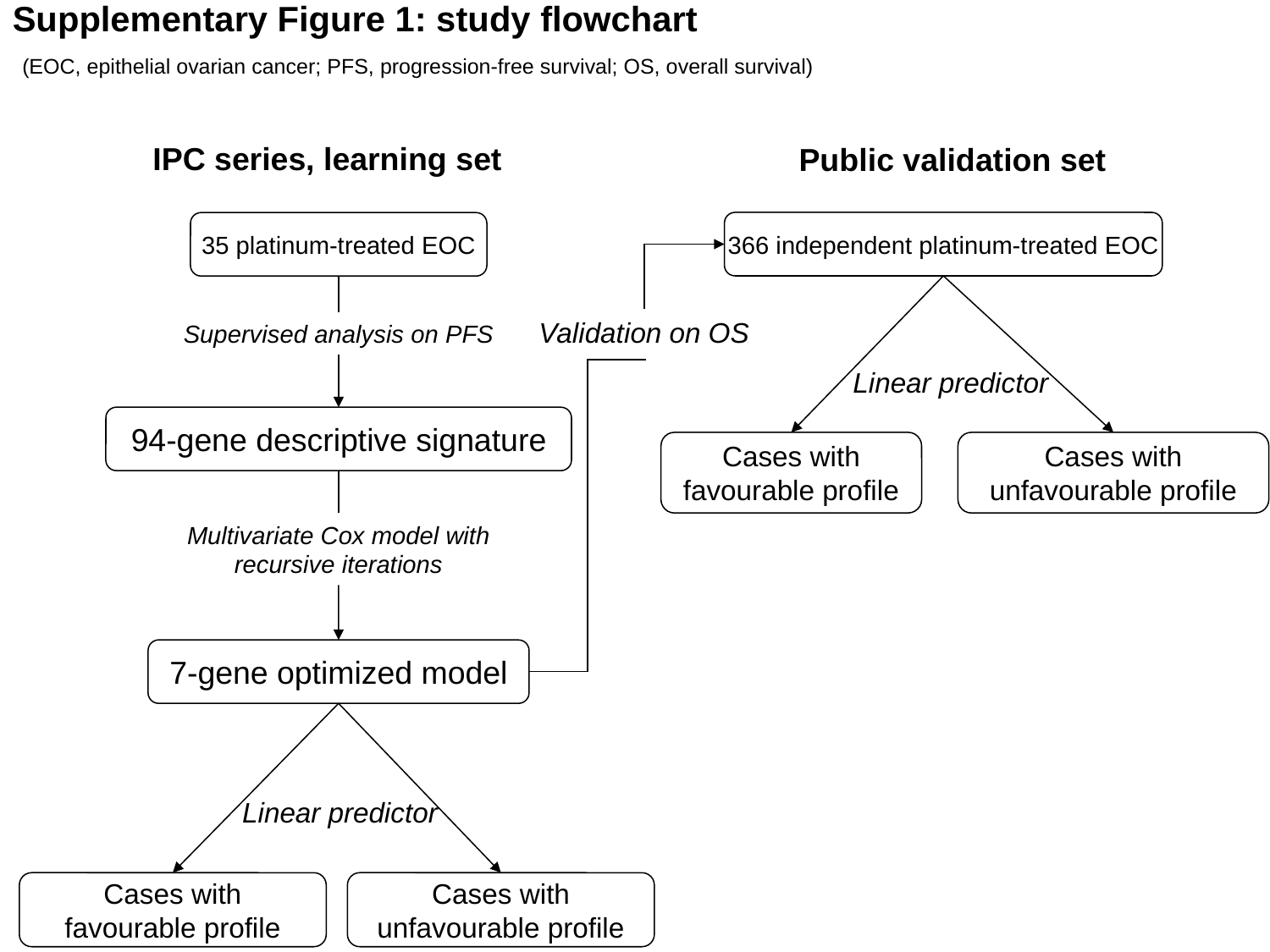

Supplementary Figure 1: study flowchart
 (EOC, epithelial ovarian cancer; PFS, progression-free survival; OS, overall survival)
IPC series, learning set
Public validation set
366 independent platinum-treated EOC
35 platinum-treated EOC
Validation on OS
Supervised analysis on PFS
Linear predictor
94-gene descriptive signature
Cases with favourable profile
Cases with unfavourable profile
Multivariate Cox model with recursive iterations
7-gene optimized model
Linear predictor
Cases with favourable profile
Cases with unfavourable profile
