# Supplementary material for: A seven-gene prognostic model for platinum-treated ovarian carcinomas
Source: Br J Cancer. 2011 Jun 7;105(2):304–11. doi: 10.1038/bjc.2011.219 (PMC3142802; doi:10.1038/bjc.2011.219)
Supplement: Supplementary materials and methods [file bjc2011219x5.doc]

**A seven-gene prognostic model for**

**platinum-treated ovarian carcinomas**

Running title: Multigene predictor for ovarian carcinomas

Renaud SABATIER, Pascal FINETTI, Julien BONENSEA,

Jocelyne Jacquemier, Jose ADELAIDE, Eric LAMBAUDIE,

Patrice VIENS, Daniel BIRNBAUM, François BERTUCCI

**SUPLEMENTARY MaterialS and methods**

**Gene expression profiling**

Total RNA isolation from frozen samples was done with the All prep DNA/RNA kit (Quiagen ™, Valencia, CA, USA). RNA quantification was done by optic density measurement (NanoDrop©); its integrity was assessed by 2100 Agilent bioanalyser (Agilent™, Palo Alto, CA, USA).

Gene expression analysis was done with Affymetrix™ Human Exon 1.0 ST arrays. Ribosomal RNA (rRNA) reduction, labelling and hybridisations were done as recommended by the manufacturer (www.Affymetrix.com). Briefly, rRNA was first reduced using biotin-labelled specific probes (LNA) and magnetic beads with streptavidine (Invitrogen™). Complementary DNA (cDNA) first-strand was then synthesised with 1µg of reduced RNA and oligo-T7 random primers and followed by second-strand synthesis. Then *in vitro* transcription associated with amplification and fragmentation (95°C during 35 minutes) produced cDNA with terminal biotin labelling. These cDNAs were then hybridized in a DNA chip with 200µl hybridization buffer at 45°C during 17 hours. Microarrays were then washed before signal amplification with biotinyled anti-streptavidin antibodies associated with streptavidin-phycoerythrin couple. Scanning was done with Affymetrix Genearray scanner and signal quantification with Affymetrix Command Console software.

**Gene expression data analysis**

We limited our expression analysis to gene level using only known and identified transcripts (Core library, Affymetrix®). Data were analysed using the PLIER (Probe Logarithmic Intensity Error) method in Expression Console (Affymetrix®). PLIER did background adjustment based on GC content, quantile normalisation and summarisation of all olignonucleotides compounding each gene, according to the Affymetrix Core library (Guide to Probe Logarithmic Intensity Error Estimation <http://www.affymetrix.com/support/technical/technotes/plier_technote.pdf>). Before analysis, a filtering step removed, from the 17,782 transcripts present on the chip, those with an expression value lower than a threshold defined by Expression Console (i.e. 171 units) in all 35 hybridisations, keeping only 11,215 genes. Analyses were then done using both unsupervised and supervised approaches.

Before **unsupervised analysis**, a second filter based on standard deviation (SD) excluded genes presenting low variations between the 35 samples. For genes with an expression value lower than background in at least one sample, SD was defined on values higher than background. We retained a total of 4,824 genes. Data were log_2_ transformed and median-centred, and then submitted to hierarchical clustering using the Cluster program (Eisen *et al*, 1998) using Pearson correlation as similarity metrics and centroid linkage clustering. Results were displayed using TreeView program (Eisen *et al*, 1998).

**Supervised analysis** (see study flowchart, **Supplementary Figure 1**), applied to the 11,215 filtered genes, aimed at identifying a multigene expression predictor for progression-free survival (PFS). First, Cox regression analysis identified genes whose expression (continuous variable) was associated with PFS (p≤0.01, Wald test). A median expression profile of progressive samples was computed from these differential genes. A correlation score (Pearson’s coefficient) of each sample with this profile was computed and used to classify samples. Two groups of samples were thus defined: an “unfavourable group” defined by a positive score, and a “favourable group” defined by a negative score. Second, we defined, from these differential genes, an optimal prognostic model (OPM). Recursive iterations were performed with a multivariate Cox model. Variables selection was done with an iterative method including two steps with leave-one-out cross-validation. The “Forward” step identified the most significant variable to classify the tumours. If its significance rate was higher than 1% and the resulting classification was better than the one from the previous model, the variable was kept. The “Backward” step took out variables one after the other one from this new model in a reverse way and evaluated all possible combinations to choose the most valuable one. This step was repeated until the model could not be improved. Once the best model was defined (Optimal Prognostic Model: OPM), a prediction score, defined by a Cox resulting linear function, was then calculated for each sample, thus defining 2 classes: the “unfavourable” class with a positive score and the “favourable” class with a negative score.

To validate the predictive performances of our OPM in independent ovarian carcinoma samples, we analysed five publicly available data sets (Berchuck *et al*, 2005; Partheen *et al*, 2006; Tothill *et al*, 2008; Berchuck *et al*, 2009; Denkert *et al*, 2009), which contained expression data of at least 30% of genes included in the model. Both expression and histoclinical data of 366 samples were collected from Duke’s data sets (Berchuck *et al*, 2005; Berchuck *et al*, 2009) available at http://data.genome.duke.edu/, and GEO database: GSE12418 (Partheen *et al*, 2006); GSE9899 (Tothill *et al*, 2008), and GSE14764 (Denkert *et al*, 2009). Three types of oligonucleotide microarrays had been used through these five sets. Before analysis, we mapped hybridisation probes across the three technological platforms, Swegene™ and Affymetrix. Affymetrix gene chips annotations were updated using NetAffx Annotation files ([www.affymetrix.com](http://www.affymetrix.com/); release from 01/12/2008). Swegene™ gene chips annotations were retrieved and updated using both SOURCE (<http://smd.stanford.edu/cgi-bin/source/sourceSearch>) and EntrezGene (Homo sapiens gene information db, release from 09/12/2008, <ftp://ftp.ncbi.nlm.nih.gov/gene/>). All probes were thus mapped based on their EntrezGene ID. When multiple probes were mapped to the same EntrezGene ID, the one with the highest variance in a particular dataset was selected to represent the gene. Data sets were then processed as follows. For the Swegene™ data set, we applied quantile normalisation to available processed data. Regarding the Affymetrix data sets, we used Robust Multichip Average (RMA) method in R using Bioconductor and associated package with the non-parametric quantile algorithm as normalisation parameter (Irizarry *et al*, 2003). RMA, applied to the raw data from all series, was done with Affymetrix Expression Console software to make a background adjustment. To test our OPM on these independent series, we first identified the common genes. We then median-centred the corresponding gene expression values within each data set (Berchuck’s sets were pooled and doubloons were excluded). The prediction score (OPM) defined two classes: “unfavourable” (positive score) and “favourable” (negative score). Regarding the prognostic analysis, the clinical outcome available in these studies was overall survival (OS). The value of time to death was available in three studies, but not in the two other studies where information was “Long survivors” if OS was superior to 7 years and “Short survivors” if inferior to 3 years in one study (Berchuck *et al*, 2005), and OS lower or higher than 5 years in the other one (Partheen *et al*, 2006).

**Statistical analysis**

Staging was assessed according to the “Fédération Internationale de Gynécologie et Obstétrique” (FIGO) classification. Surgery was defined as optimal if residual disease was equal or smaller than 1 cm, and as suboptimal when residual disease was larger than 1 cm. In most of validation series the only surgical result available was the presence or absence of residual disease; this point was thus tested to evaluate prognostic robustness of our model. At the end of chemotherapy, patients were considered as showing clinical complete response (CCR) if they did not have any clinical and radiological sign of disease and if they had serum CA125 normalisation. Pathological complete response (pCR) was defined as the absence of macroscopic or microscopic residual disease during second look surgery. Follow-up was calculated from the date of diagnosis to the date of last news for surviving patients. Progression-free survival (PFS) in the IPC series was calculated from the end of chemotherapy to the date of disease progression. In the IPC and validation sets, OS was calculated from the date of diagnosis to the date of death from any cause.

Correlation between groups of samples and histoclinical data was assessed by a Fisher’s exact test for binary values and a Mann-Whitney test for continuous variables. Kaplan-Meier analyses with the log-rank test were used to estimate and compare PFS and OS variations between the groups. Prognostic analyses were done in an univariate way with the usual histoclinical criteria (age, grade, FIGO stage, taxane use when available, surgical status) and the OPM-based classification. Independent prognostic factors were then identified with multivariate analysis considering variables with a *p*-value lower than 0.05 in univariate analysis. In our IPC set, the survival endpoint was PFS analysed as a continuous variable (progression date available). In the validation set, we used OS (PFS was not available in all series). Kaplan-Meier method was used when time to death was available (Tothill *et al*, 2008; Berchuck *et al*, 2009; Denkert *et al*, 2009) and statistical difference was assessed with the log-rank test. Significant values were kept with a 5% threshold. All statistical analyses were done in R (2.6.1) and its associated packages.

**REFERENCES**

Berchuck A, Iversen ES, Lancaster JM, Pittman J, Luo J, Lee P, Murphy S, Dressman HK, Febbo PG, West M, Nevins JR, Marks JR (2005) Patterns of gene expression that characterize long-term survival in advanced-stage serous ovarian cancers. *Clin Cancer Res* **11**: 3686-3696

Berchuck A, Iversen ES, Luo J, Clarke JP, Horne H, Levine DA, Boyd J, Alonso MA, Secord AA, Bernardini MQ, Barnett JC, Boren T, Murphy SK, Dressman HK, Marks JR, Lancaster JM (2009) Microarray analysis of early stage serous ovarian cancers shows profiles predictive of favorable outcome. *Clin Cancer Res* **15**: 2448-2455

Denkert C, Budczies J, Darb-Esfahani S, Györffy B, Sehouli J, Könsgen D, Zeillinger R, Weichert W, Noske A, Buckendahl AC, Müller BM, Dietel M, Lage H (2009) A prognostic gene expression index in ovarian cancer - validation across different independent data sets. *J Pathol* **218**: 273-280

Eisen MB, Spellman PT, Brown PO, Botstein D (1998) Cluster analysis and display of genome-wide expression patterns. *Proc Natl Acad Sci USA* **95**: 14863–14868

Irizarry RA, Hobbs B, Collin F, Beazer-Barclay YD, Antonellis KJ, Scherf U, Speed TP (2003) Exploration, normalization, and summaries of high density oligonucleotide array probe level data. *Biostatistics* **4**: 249-264

Partheen K, Levan K, Osterberg L, Horvath G (2006) Expression analysis of stage III serous ovarian adenocarcinoma distinguishes a sub-group of survivors. *Eur J Cancer* **42**:2846-2854

Tothill RW, Tinker AV, George J, Brown R, Fox SB, Lade S, Johnson DS, Trivett MK, Etemadmoghadam D, Locandro B, Traficante N, Fereday S, Hung JA, Chiew YE, Haviv I; Australian Ovarian Cancer Study Group, Gertig D, DeFazio A, Bowtell DD (2008) Novel molecular subtypes of serous and endometrioid ovarian cancer linked to clinical outcome. *Clin Cancer Res* **14**: 5198-5208
